# Improved 2,3-Butanediol Production Rate of Metabolically Engineered *Saccharomyces cerevisiae* by Deletion of *RIM15* and Activation of Pyruvate Consumption Pathway

**DOI:** 10.3390/ijms242216378

**Published:** 2023-11-15

**Authors:** Masahiko Sugimura, Taisuke Seike, Nobuyuki Okahashi, Yoshihiro Izumi, Takeshi Bamba, Jun Ishii, Fumio Matsuda

**Affiliations:** 1 Department of Bioinformatics Engineering, Graduate School of Information Science and Technology, Osaka University, 1-5 Yamadaoka, Suita 565-0871, Osaka, Japan; 2 Division of Metabolomics/Mass Spectrometry Center, Medical Research Center for High Depth Omics, Medical Institute of Bioregulation, Kyushu University, 3-1-1 Maidashi, Higashi-ku, Fukuoka 812-8582, Fukuoka, Japan; 3 Graduate School of Science, Technology and Innovation, Kobe University, 1-1 Rokkodai, Nada, Kobe 657-8501, Hyogo, Japan

**Keywords:** *Saccharomyces cerevisiae*, 2,3-butanediol, *RIM15*, metabolome analysis, Gibbs free energy change, metabolic engineering

## Abstract

*Saccharomyces cerevisiae* is a promising host for the bioproduction of higher alcohols, such as 2,3-butanediol (2,3-BDO). Metabolically engineered *S. cerevisiae* strains that produce 2,3-BDO via glycolysis have been constructed. However, the specific 2,3-BDO production rates of engineered strains must be improved. To identify approaches to improving the 2,3-BDO production rate, we investigated the factors contributing to higher ethanol production rates in certain industrial strains of *S. cerevisiae* compared to laboratory strains. Sequence analysis of 11 industrial strains revealed the accumulation of many nonsynonymous substitutions in *RIM15*, a negative regulator of high fermentation capability. Comparative metabolome analysis suggested a positive correlation between the rate of ethanol production and the activity of the pyruvate-consuming pathway. Based on these findings, *RIM15* was deleted, and the pyruvate-consuming pathway was activated in YHI030, a metabolically engineered *S. cerevisiae* strain that produces 2,3-BDO. The titer, specific production rate, and yield of 2,3-BDO in the test tube-scale culture using the YMS106 strain reached 66.4 ± 4.4 mM, 1.17 ± 0.017 mmol (g dry cell weight h)^−1^, and 0.70 ± 0.03 mol (mol glucose consumed)^−1^. These values were 2.14-, 2.92-, and 1.81-fold higher than those of the vector control, respectively. These results suggest that bioalcohol production via glycolysis can be enhanced in a metabolically engineered *S. cerevisiae* strain by deleting *RIM15* and activating the pyruvate-consuming pathway.

## 1. Introduction

*Saccharomyces cerevisiae* is a promising host for the bioproduction of higher alcohols, such as 2,3-butanediol (2,3-BDO) [1,2,3]. 2,3-BDO bioproduction by *S. cerevisiae* has garnered notable interest owing to its versatility as a raw material for pharmaceuticals, cosmetic intermediates, and various high-value chemicals [4,5]. Moreover, 1,3-butadiene can be synthesized from 2,3-BDO through dehydration and serves as a precursor for both synthetic rubber and liquid fuels [2,6]. In *S. cerevisiae*, the Embden–Meyerhof pathway catabolizes glucose to pyruvate. The Ehrlich pathway converts pyruvate to ethanol via pyruvate decarboxylase (PDC) and alcohol dehydrogenase (ADH). Metabolically engineered *S. cerevisiae* strains that produce 2,3-BDO were constructed by introducing an artificial three-step pathway from pyruvate to 2,3-BDO [6,7,8,9,10,11,12,13,14]. In addition, 2,3-BDO production is elevated by disrupting competing pathways and solving the cofactor imbalance [6,7,8,9,10,11,12,13,14,15,16,17]. Fed-batch cultivation of the most advanced strains achieved 2,3-BDO production with very high titers [8,9]. However, the 2,3-BDO production rates of these engineered strains were less than one-tenth of the specific ethanol production rates of non-engineered *S. cerevisiae* [7,9,13].

To identify approaches for improving the 2,3-BDO production rate, we focused on the fact that some industrial strains of *S. cerevisiae* exhibit higher ethanol production rates than laboratory strains [15,18]. The molecular mechanisms underlying the high ethanol production rate of non-engineered industrial *S. cerevisiae* strains could be applied to improve the 2,3-BDO production rate of engineered strains. The high fermentation capability of industrial strains could be attributed to the activation or inactivation of some metabolic reactions in the central carbon metabolism. To identify the responsible reactions, metabolome analysis of central metabolism provides comprehensive concentration data for intracellular metabolites [19]. The metabolite concentration data are also available to calculate the Gibbs free energy change (Δ*G*′) levels of each reaction or pathway [20,21,22]. Target reactions for metabolic engineering can be estimated from a correlation analysis between intracellular metabolite state data and the ethanol production rate.

Furthermore, the high fermentation capability of the sake (Kyokai) strain is attributable to a loss-of-function mutation in *RIM15*, which encodes a protein kinase involved in cell proliferation in response to nutrients [16,17,23]. The name RIM15 was derived from “Regulator of IME2 (Inducer of MEiosis)” [24]. Additionally, ethanol fermentation by a laboratory strain was improved by deleting *RIM15* [16,17]. Disruption of *RIM15* may contribute to increased tolerance to heavy metals and improve the glycerol assimilation ability of *S. cerevisiae* [25,26]. However, the effect of the *RIM15* deletion on 2,3-BDO production remains to be comprehensively investigated.

In this study, we performed a comparative analysis of 11 industrial strains of *S. cerevisiae,* including laboratory, sake, wine, bread, and beer strains, with different fermentation rates. Sequence analysis of *RIM15* and metabolic analysis indicated that the deletion of *RIM15* and activation of the pyruvate- and NADH-consuming pathways are promising strategies for improving 2,3-BDO production. These strategies were tested in a wild-type laboratory strain and implemented in a metabolically engineered strain for 2,3-BDO production. We found that *RIM15* deletion and activation of the pyruvate- and NADH-consuming pathways improved the 2,3-BDO production rate.

## 2. Results

### 2.1. Culture Profiles and RIM15 Mutations in 11 Industrial Diploid Strains

**Figure 1:**
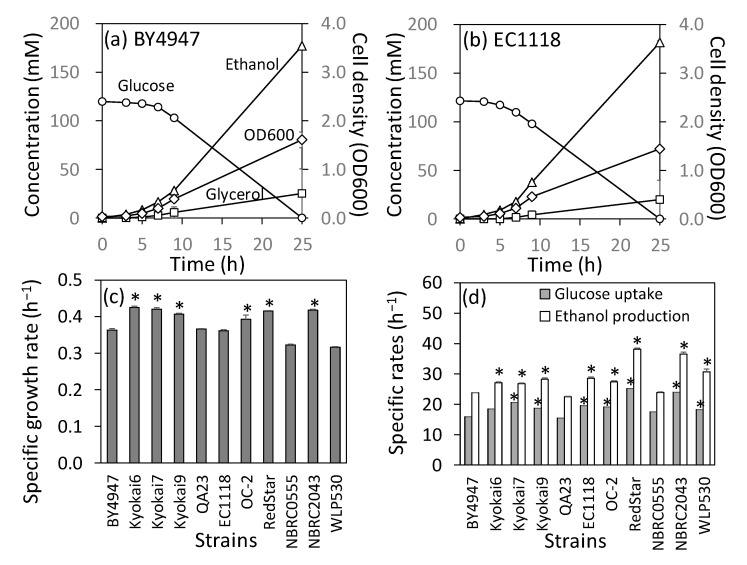
Culture profiles of 11 industrial diploid yeast strains. Culture profiles were obtained from one laboratory (BY4947, a diploid of S288C), three sake (Kyokai6, Kyokai7, and Kyokai9), three wine (QA23, EC1118, and OC-2), three bread (RedStar, NBRC0555, and NBRC2043), and one beer (WLP530) strains. (**a**) Culture profile of BY4947. (**b**) Culture profile of EC1118. (**c**) Specific growth rates of the 11 strains. (**d**) Specific rates of glucose uptake and ethanol production. The specific rates were determined based on data from 3 to 9 h. All cultures were performed in triplicate. Error bars indicate the standard deviation. Asterisks indicate the results of the two-sided *t*-test (*p* < 0.05, *n* = 3).

Culture profiles of 11 industrial diploid yeast strains were obtained from flask-scale batch cultivation in a synthetic medium under aerobic conditions (Figure 1). One laboratory (BY4947, a diploid of S288C), three sake (Kyokai6, Kyokai7, and Kyokai9), three wine (QA23, EC1118, and OC-2), three bread (RedStar, NBRC0555, and NBRC2043), and one beer (WLP530) strains were used. For BY4947, exponential growth occurred 3–9 h after culture initiation (Figure 1a). The specific rates of cell growth and ethanol production were 0.363 ± 0.005 h^−1^ and 23.8 ± 0.1 mmol (g dry cell weight [DCW] h)^−1^, respectively. Figure 1b shows the culture profile of the EC1118 strain used for wine production. Glucose consumption and ethanol production of EC1118 at 3–9 h were higher than those in BY4947. The specific ethanol production rate of EC1118 was 28.5 ± 0.4 mmol (g DCW h)^−1^, 19% higher than that of BY4947.

A comparison of the 11 strains showed that the three sake strains (Kyokai6, Kyokai7, and Kyokai9) tended to have higher specific growth, glucose consumption, and ethanol production rates than those of BY4947 (Figure 1c,d). Other strains showed elevated specific rates of glucose consumption and ethanol production, except for QA23 and NBRC0555 (Figure 1d). However, increased specific growth rates were observed only in OC-2, RedStar, and NBRC2043 (Figure 1c). Particularly, the specific ethanol production rates of baker’s yeast, RedStar and NBRC2043, reached 38.2 ± 0.3 and 36.5 ± 0.6 mmol (g DCW h)^−1^, which were 160% and 153% of the rate observed for BY4947, respectively (Figure 1d). These results are generally consistent with those reported in previous studies [18].

A frameshift mutation at nucleotide position 5068 in *RIM15* (named A1686fs) results in the production of defective Rim15p, leading to the high fermentation performance of Kyokai6, 7, and 9 strains [23,27]. The fermentability of a laboratory strain also improved when *RIM15* was deleted [16,17]. To investigate mutations in other industrial strains, sequence analyses of *RIM15* were conducted for the 11 strains. The results confirmed that *RIM15* in the Kyokai6, 7, and 9 strains contained the A1686fs mutation and four nonsynonymous substitutions (Tables S1 and S2). Furthermore, the other wine, bread, and beer strains accumulated seven to nine nonsynonymous substitutions, although they did not possess the A1686fs mutation. Among them, Rim15p in the seven non-sake strains contained the E607D, T609S, and T723S substitutions (Table S2). Although further confirmatory studies are needed, the accumulation of nonsynonymous substitutions may impair Rim15p function.

### 2.2. Correlation between the Intracellular Metabolite State and Ethanol Production Rate

Metabolomic analysis of intracellular metabolites was performed to examine the metabolic state shift caused by ethanol production activation. All strains were cultured under the aforementioned conditions. Metabolites were extracted from late log phase cells (optical density at 600 nm (OD_600_) = 1.0). Metabolite concentrations were determined using liquid chromatography-tandem mass spectrometry-based metabolome analysis using a stable isotope dilution method. The intracellular concentrations of 84 metabolites, including ATP, NADH, sugar phosphates, amino acids, and nucleic acids, were measured in the 11 strains (Data S1). Using the metabolite concentration data, changes in Gibbs free energy change (Δ*G*′) were calculated for 26 metabolic reactions or pathways (Data S2). The calculation was based on several assumptions, such as equal metabolite concentrations in the mitochondria and cytosol, identical intracellular and extracellular concentrations of glucose and ethanol, and fixed concentrations of phosphate and CO_2_ (see Materials and Methods for details).

**Figure 2:**
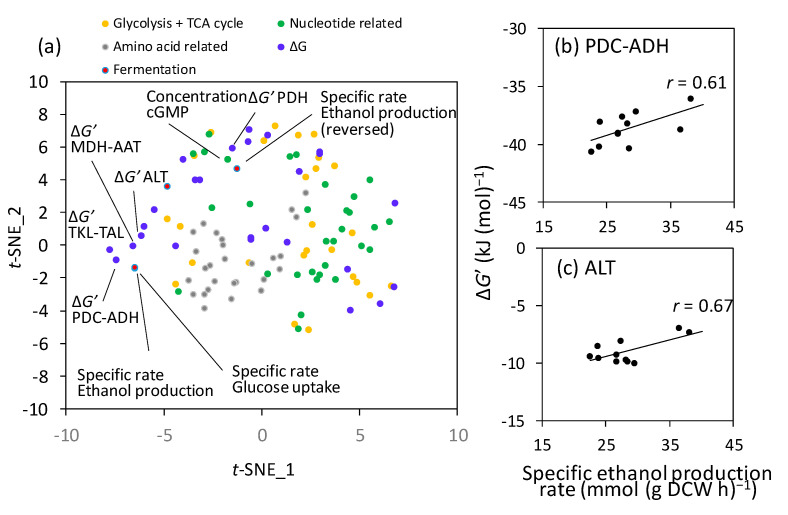
Comparison of metabolite states among 11 industrial yeast strains. (**a**) Results of *t*-distributed stochastic neighbor embedding (*t*-SNE). A data matrix were analyzed, including 84 metabolite concentrations, 26 Δ*G*′ values, and four specific rates. (**b**,**c**) Scatter plots between the specific ethanol production rate (*x*-axis) and levels of (**b**) Δ*G*′(PDC-ADH) and (**c**) Δ*G*′(ALT). The correlation coefficients (*r*) are shown in the panels. AAT, aspartate aminotransferase; ADH: alcohol dehydrogenase; ALT: alanine transaminase; MDH, malate dehydrogenase; PDC: pyruvate decarboxylase; PDH: pyruvate dehydrogenase; TCA: tricarboxylic acid; *t*-SNE: *t*-distributed stochastic neighbor embedding. The definition of reaction names is shown in Table S3.

Subsequently, a data matrix of the 84 metabolite concentrations, 26 Δ*G*′ values, and four specific rates was analyzed using the *t*-distributed stochastic neighbor embedding (*t*-SNE) method (Figure 2a). In addition to the specific rates of cell growth, glucose consumption, and ethanol production, the reversed specific ethanol production rate was used to detect negative correlations. The results of *t*-SNE showed that the specific rates of ethanol production and glucose consumption were located at very similar positions (Figure 2a), reflecting a close correlation between these rates (Figure 1d). Additionally, the Δ*G*′ of four pathways, namely the Δ*G*′(PDC-ADH), Δ*G*′(alanine transaminase (ALT)), Δ*G*′(transketolase (TKL)-transaldolase (TAL)), and Δ*G*′(malate dehydrogenase (MDH)-aspartate aminotransferase (AAT)), were close to the specific rate of ethanol production (Figure 2a, the definition of reaction names is shown in Table S3). Moreover, Δ*G*(pyruvate dehydrogenase (PDH)) and cGMP were located near the reversed specific ethanol production rate. Hierarchical clustering analysis using a smaller data matrix, including 26 Δ*G*′ values and four specific rates, confirmed that the specific ethanol production rate, Δ*G*′(PDC-ADH), Δ*G*′(ALT), Δ*G*′(TKL-TAL), and Δ*G*′(MDH-AAT) were in the same cluster (Figure S1).

These results suggest that pyruvate consumption reactions may have been activated in strains with improved specific ethanol production rates because the PDC-ADH and ALT pathways consume pyruvate as a substrate. The specific ethanol production rate showed positive correlations with Δ*G*′(PDC-ADH) (*r* = 0.61; Figure 2b) and Δ*G*′(ALT) (*r* = 0.67; Figure 2c). The PDC-ADH pathway is an ethanol biosynthetic pathway from pyruvate, and its activation likely elevates ethanol production. The ALT pathway synthesizes alanine from pyruvate and supplies amino acids for cell growth.

### 2.3. Elevation of Ethanol Production in the Laboratory Strain by Activating the Pyruvate-Consuming Reaction

**Figure 3:**
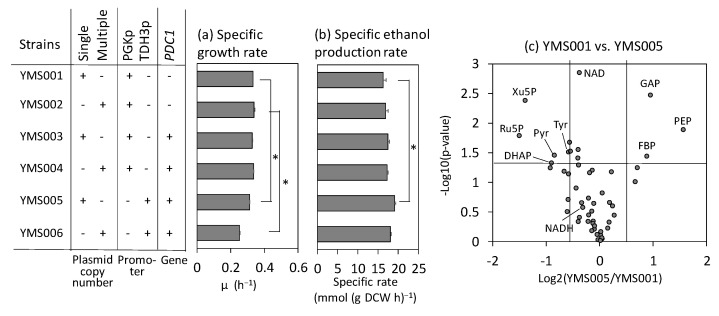
Effect of *PDC1* overexpression on ethanol production in laboratory strains. (**a**) Specific growth rate. (**b**) Specific ethanol production rate determined from 3–9 h data. The data are presented as the mean ± standard deviation of three independent transformants (*n* = 3 each). Asterisks indicate the results of the two-sided *t*-test (*p* < 0.05, *n* = 3). (**c**) Comparison of the metabolic profiles of YMS005 and vector control in a volcano plot. Metabolome data were obtained from cells at the mid-log phase (9 h, *n* = 3). DHAP: dihydroxyacetone phosphate; FBP: fructose-1,6-bisphosphate; GAP: glyceraldehyde 3-phosphate; PEP: phosphoenolpyruvate; Pyr: pyruvate; Ru5P: ribulose-5-phosphate; Tyr: tyrosine.

The results of the metabolic analyses described previously suggest that the activation of the pyruvate-consuming reaction increases the ethanol production rate. To confirm this finding, *PDC1* from *S. cerevisiae* was overexpressed in the haploid laboratory yeast strain (YPH499). *PDC1* encodes a major isoform of PDC, the initial enzyme involved in ethanol biosynthesis from pyruvate [28]. Strains YMS003 and YMS004 were constructed by introducing single- and multi-copy plasmids expressing *PDC1* under the control of the PDC1p promoter, respectively. Flask-scale batch cultivation showed that the specific rates of ethanol production and cell growth of the YMS003 and YMS004 strains were similar to those of the vector control strains (YMS001 and YMS002), likely because of insufficient overexpression of *PDC1* (Figure 3a,b).

To further overexpress *PDC1*, the YMS005 and YMS006 strains were constructed by replacing the PDC1p promoter and PDC1t terminator with the stronger TDH3p and TDH3t, respectively. *PDC1* overexpression resulted in an increased burden in the YMS006 strain, which contains the multi-copy type plasmid, as the specific growth rate decreased by 25% (Figure 3a) despite a 7% increase in the specific ethanol production rate (Figure 3b). Severe growth defects caused by PDC1p overexpression were alleviated in the YMS005 strain with the single-copy-type plasmid (Figure 3a). In this strain, the specific ethanol production rate increased by 17% compared with that of the empty vector strain (Figure 3b).

To investigate the effects of *PDC1* overexpression on intracellular metabolism, metabolites were extracted from YMS001 and YMS005 cells after 9 h and subjected to metabolomic analyses (Data S3). The volcano plot revealed that the pyruvate content decreased significantly following the overexpression of *PDC1*, whereas NAD and NADH levels did not change significantly (Figure 3c). Moreover, the levels of key metabolites involved in glycolysis, including glyceraldehyde 3-phosphate, phosphoenolpyruvate, and fructose-1,6-bisphosphate, were elevated upon *PDC1* overexpression. Elevated fructose-1,6-bisphosphate levels are a signature of high glycolytic flux and temporal activation of glucose uptake [29,30,31]. These results demonstrate that the specific ethanol production rate can be elevated by the activation of the pyruvate-consuming reaction, as predicted by metabolome analysis.

### 2.4. Implementation of RIM15 Deletion and Enhanced Pyruvate Consumption in a Metabolically Engineered S. cerevisiae Strain Producing 2,3-Butanediol

**Figure 4:**
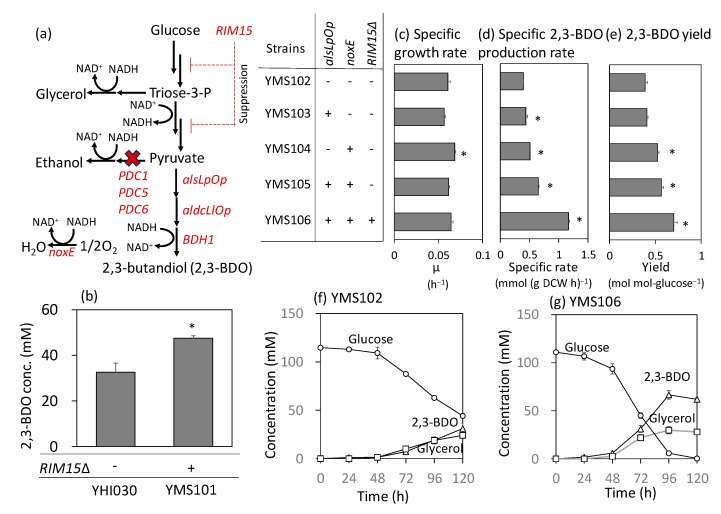
Implementation of *RIM15* deletion and activation of the pyruvate-consuming pathway in a metabolically engineered *S. cerevisiae* strain producing 2,3-butanediol (2,3-BDO). All strains were constructed from the YHI030 strain developed by [13]. (**a**) The metabolic pathway engineered in this study. (**b**) Effect of the *RIM15* deletion on 2,3-BDO production. The 2,3-BDO concentrations in the flask-scale culture were determined at 72 h. (**c**–**e**) Specific growth rate (**c**), specific 2,3-BDO production rate (**d**), and 2,3-BDO yield (**e**) of metabolically engineered strains in the test tube-scale culture. Specific rates were determined from 24–72 h data. (**f**,**g**) Culture profiles of engineered strains in the test tube-scale culture. Culture profiles of YMS106 (**f**) and YMS106 (**g**) are shown. The data are presented as the mean ± standard deviation of three independent transformants (*n* = 3 each). Asterisks indicate the results of the two-sided *t*-test (*p* < 0.05, *n* = 3).

Findings from industrial strains were implemented using a metabolically engineered *S. cerevisiae* strain that produces 2,3-BDO. In our previous study, the YHI030 strain producing 2,3-BDO was constructed from the YPH499 strain by introducing the *MTH1*-ΔT mutation, along with the deletion of *PDC1*, *PDC5*, and *PDC6*. Additionally, laboratory evolution was employed to improve the growth rate, and three genes (*alsLpOp*, *aldcLlOp*, and *BDH1*) necessary for the biosynthesis of 2,3-BDO from pyruvate were introduced (Figure 4a) [13]. The YHI030 strain did not produce ethanol as all pyruvate decarboxylase genes had been deleted in a previous study (Figure 4a) [13]. Moreover, the YHI030 strain produced 2,3-BDO with a yield of 0.41 g g^−1^ glucose in fed-batch cultivation using synthetic dextrose (SD) medium [13]. This level is similar to those of other strains reported previously in fed-batch cultivation using a rich medium (0.41–0.48 g g^−1^ glucose) [7,9].

First, the open reading frame (ORF) of *RIM15* was deleted from YHI030 to construct the YMS101 strain. Strain YHI030 produced 33.5 ± 4.0 mM (3.0 ± 0.3 g L^−1^) of 2,3-BDO after 72 h, following cultivation at the flask scale with an initial OD_600_ of 0.5 (Figure 4b). The YMS101 strain produced 1.4-fold more 2,3-BDO, confirming that the loss-of-function of *RIM15* also improved 2,3-BDO production (Figure 4b).

Second, the pyruvate-consuming pathway was activated by the additional activation of acetolactate synthase, which is responsible for the first step of the 2,3-BDO synthesis pathway (Figure 4a). The YMS103 strain was constructed from YHI030 by overexpressing *alsLpOp*, a codon-optimized ORF of *alsLp* derived from *Lactobacillus plantarum* [13]. The strain was cultured at the test tube scale with an initial OD_600_ of 0.1 to investigate 2,3-BDO production (Figure 4c–e). The specific production rate and yield of YMS103 increased by 5% and 10%, respectively, compared with those of the empty vector strain, YMS102. However, the specific growth rate decreased by 7% (Figure 4c), likely due to the NADH imbalance associated with 2,3-BDO production. Because the introduction of NADH oxidase relaxes this imbalance [6,10], a codon-optimized ORF of *noxE* from *Lactococcus lactis* was expressed in YHI030 (Figure 4a). The specific growth rate of the constructed strain YMS104 was 107% of that of the vector control, whereas the specific production rate and yield of 2,3-BDO were 128% and 135%, respectively (Figure 4c–e). Furthermore, a YMS105 strain combined with *alsLpOp* overexpression and *noxE* expression was constructed. The specific production rate and yield of 2,3-BDO were 164% and 145%, respectively, as compared with those of the vector control (Figure 4d,e).

Finally, the *RIM15* deletion was introduced into the YMS105 strain. Figure 4f,g show the culture profiles of the empty vector (YMS102) and constructed (YMS106) strains at the test tube scale. Some glucose remained in the YMS102 strain even after 120 h (Figure 4f), whereas the YMS106 strain completely consumed glucose after 96 h (Figure 4g). The maximal 2,3-BDO titer increased from 31.1 ± 1.8 to 66.4 ± 4.4 mM, which improved the yield of 2,3-BDO from 0.39 ± 0.03 to 0.70 ± 0.03 mol (mol glucose)^−1^. Particularly, the specific rate of 2,3-BDO production reached 1.17 ± 0.017 mmol (g DCW h)^−1^, which was approximately 3-fold higher than that of YMS102 (0.40 ± 0.004 mmol (g DCW h)^−1^) (Figure 4d). The productivity also increased from 0.25 ± 0.02 to 0.69 ± 0.05 mmol (l h)^−1^. These results indicate that 2,3-BDO production can be enhanced by combining the deletion of *RIM15* with the activation of the pyruvate consumption pathway.

## 3. Discussion

In the present study, we performed comparative genetic and metabolomic analyses of 11 industrial and laboratory strains of *S. cerevisiae*. The engineering targets identified from the analyses were used to improve the 2,3-BDO production of metabolically engineered *S. cerevisiae* constructed in our previous study (YHI030) [13]. Sequence analysis of *RIM15* confirmed that the A1686fs frameshift was present only in the sake strain and that other industrial strains accumulated nonsynonymous substitutions at seven to nine sites, which may impair the function of the Rim15p protein (Table S2). Although the role of these mutations requires further analysis, additional deletion of the *RIM15* strain improved the production rate and yield of 2,3-BDO in YHI030 (Figure 4b), suggesting that *RIM15* deletion is a promising tool for metabolic engineering.

Furthermore, metabolome analyses indicated that the activation of the pyruvate-consuming pathway could be an additional target (Figure 2). Variation in the specific ethanol production rates among the 11 strains was positively correlated with the Δ*G*′ levels of the PDC-ADH pathway producing ethanol from pyruvate (Figure 2c). This result suggests that further activation of the pyruvate-consuming pathway would increase the ethanol production rate in the ethanol-producing laboratory strain and increase the 2,3-BDO production rate in the metabolically engineered strain generating 2,3-BDO (YHI030). To test this hypothesis, we initially verified that the ethanol production rate could be increased by additional overexpression of *PDC1* in the ethanol-producing laboratory strain (Figure 3). Similar increases in specific ethanol production rates have been reported for a PDC1-overexpressing strain derived from CEN.PK113-7D [32]. However, the phenotype was subtle and condition-dependent, as the copy number and promoter activity for *PDC1* expression affected the production rate (Figure 3). Specific ethanol production rates improve only over a limited range of dilution rates [32]. One reason for condition dependency is post-translational regulation. Pyruvate decarboxylase activity in *S. cerevisiae* is regulated by the phosphorylation of the Ser/Thr protein phosphatase Sit4p [33]. Because of the post-translational regulation, the expression levels of Pdc1p did not correlate with the ethanol production rate. For example, the expression levels of Pdc1p protein in Kyokai7 and RedStar were lower than those in BY4947 during aerobic batch cultivation in SD medium [18].

Based on the confirmation of this hypothesis, we activated the 2,3-BDO biosynthesis pathway from pyruvate in the YHI030 strain by overexpressing *alsLpOp* and *noxE* (Figure 4). Notably, the suppression of ethanol biosynthesis was impossible due to the inability of the YHI030 strain to produce ethanol resulting from the deletion of all pyruvate decarboxylase genes [13]. However, the additional overexpression of enzymes was anticipated to activate the 1,3-BDO pathway in pyruvate. This is because post-translational regulation in *S. cerevisiae* is unlikely to control enzymes derived from other organisms. Using the YHI030 as a parental strain, this study demonstrated that activation of the pyruvate-consuming pathway improved 2,3-BDO production by overexpressing *alsLpOp* from *L. plantarum* and *noxE* from *L. lactis*. The specific production rate and yield of 2,3-BDO in the YMS105 strain combined with *alsLpOp* overexpression and *noxE* expression were 164% and 145%, respectively, as compared with those in the vector control (Figure 4d,e).

This study also demonstrated that the combined *RIM15* deletion and activation of the pyruvate-consuming pathway effectively improved 2,3-BDO production. The titer, specific production rate, and yield of 2,3-BDO of the YMS106 strain reached 66.4 ± 4.4 mM, 1.17 ± 0.017 mmol (g DCW h)^−1^, and 0.70 ± 0.03 mol (mol glucose consumed)^−1^, respectively, marking a 2.14-, 2.92-, and 1.81-fold increase compared with those in the vector control (YMS102) (Figure 4d–g). In *RIM15*-deleted strains, the pathway from glucose to pyruvate (the Embden–Meyerhof pathway) was activated by the native regulation mechanism of *S. cerevisiae* (Figure 4a). However, this regulatory mechanism was not effective in the artificial biosynthetic pathway from pyruvate to 2,3-BDO. Therefore, the combined implementation of *RIM15* deletion and activation of the 2,3-BDO biosynthesis pathway activated the entire 2,3-BDO production pathway from glucose.

The previously reported yields of 2,3-BDO by metabolically engineered *S. cerevisiae* strains were 0.11 g g-glucose^−1^ in batch culture [5], 0.42 g g-glucose^−1^ in fed-batch culture [34], and 0.54 g g-glucose^−1^ in continuous culture [34]. The theoretical maximum yield of 2,3-BDO from glucose is 0.5 g g-glucose^−1^. The 2,3-BDO yield demonstrated in this study was 0.70 ± 0.03 mol mol-glucose^−1^, equivalent to 0.35 g g-glucose^−1^. The yield can be improved by optimizing the aeration conditions and applying a fed-batch culture using a rich medium [13]. However, there is still potential for improvement in the specific 2,3-BDO production rate of the engineered strain when compared to the specific ethanol production rate of the non-engineered strains. For instance, the specific ethanol production rate of BY4749 (the laboratory strain) shown in Figure 1 was 23.8 ± 0.1 mmol (g DCW h)^−1^, equivalent to the specific 2,3-BDO production rate of 11.9 ± 0.1 mmol (g DCW h)^−1^. This is because two molecules of ethanol and one molecule of 2,3-BDO can be synthesized from one glucose molecule. The best specific 2,3-BDO production rate demonstrated in this study (YMS106 in Figure 4d) was 1.17 ± 0.017 mmol (g DCW h)^−1^, which was still only 10% of the ethanol production capacity.

To further improve the metabolically engineered *S. cerevisiae* strains producing 2,3-BDO, activation of glycolysis by *RIM15* deletion is expected to be useful. For example, 2,3-BDO production yield improves when glycerol coproduction is suppressed [7] and ATP wasting is elevated [35]. The δ integration method was employed to activate the artificial pathway from pyruvate to 2,3-BDO [9]. Combining these methods with the activation of the Embden–Meyerhof pathway by *RIM15* deletion should further improve the production of 2,3-BDO. The *RIM15* deletion is also expected to be effective in the bioproduction of other target compounds from pyruvate [36,37]. Furthermore, homologous genes of *RIM15* are widely distributed in many fungi. The expression of the *UGP1* gene encoding UDPG pyrophosphorylase, which is required for polysaccharide production in a yeast-like fungus (*Aureobasidium melanogenum*), is regulated by a similar mechanism downstream of the *RIM15* signaling pathway in *S. cerevisiae* [38,39].

This study demonstrates that modification of the native regulatory mechanism in *S. cerevisiae* by *RIM15* deletion effectively improves 2,3-BDO production. Further research is required to enhance our understanding of the regulatory mechanisms governing the Embden–Meyerhof pathway as most metabolically engineered *S. cerevisiae* strains use this pathway to catabolize glucose. The metabolic analysis of a strain that overexpresses *RIM15* will help elucidate the comprehensive functions of Rim15p in regulating the Embden–Meyerhof pathway. A more comprehensive comparative analysis of the metabolism of *S. cerevisiae*, given its various fermentation capabilities, would unveil the intricate regulatory mechanisms. Such an investigation can offer valuable insights and provide a useful approach for the metabolic engineering of *S. cerevisiae*.

## 4. Materials and Methods

### 4.1. Strains and Culture Condition

*Saccharomyces cerevisiae* strains and plasmids used in this study are listed in Table 1. A yeast extract-peptone-dextrose (YPD) medium (1% Bacto yeast extract, 2% Bacto peptone, and 2% glucose) and an SD medium (2% glucose and 0.67% yeast nitrogen base without amino acids) were used. To culture the metabolically engineered strains, amino acids and nucleic acids were added, as necessary. SDGal medium supplemented with 2% galactose rather than glucose was used for genome editing.

For the preculture of diploid industrial strains and engineered strains producing ethanol, a single colony on the YPD plate was inoculated into a test tube containing 5 mL of YPD medium and cultured overnight at 30 °C with shaking at 150 rpm. For preculture, the cells were transferred to 50 mL of SD medium in a 200 mL baffled flask and cultured for 16 h at 30 °C with shaking at 120 rpm. This preculture solution was inoculated into 50 mL of SD medium in a 200 mL baffled flask with an initial OD_600_ of 0.05 and cultured under the same conditions as the preculture. To culture the 2,3-BDO-producing strains derived from YHI030, a single colony was inoculated into a test tube containing 5 mL of SD medium and cultured for three days under shaking conditions (30 °C, 150 rpm). For the preculture and main culture, the cells were transferred into 5 mL of SD medium in a test tube and cultured under the same conditions. The initial OD_600_ of the primary culture was 0.1 or 0.5. The OD_600_ was measured using a spectrophotometer (UV-1700, Shimadzu, Kyoto, Japan).

### 4.2. Measurement of Extracellular Metabolites

The concentrations of ethanol, glucose, and glycerol in the filtrated medium (0.45 µm pore size Cosmonice filter W, Nacalai Tesque, Kyoto, Japan) were measured as previously described using high-performance liquid chromatography (Prominence, Shimadzu, Kyoto, Japan) [13]. To measure the 2,3-BDO concentration, 50 µL of the filtered medium was diluted with an equal amount of 0.1% 3-methyl-1-butanol solution and measured using a gas chromatograph (GC-2025, Shimadzu) as described previously.

### 4.3. Metabolome Analysis

Metabolome analysis was conducted as described previously, with slight modifications [20]. Briefly, *S. cerevisiae* cells were collected from culture broth (approximately OD600 × mL = 8.0) using the filter method (polytetrafluorethylene membrane filter: 0.45 µm pore size, 47 mm filter diameter; Omnipore, Merck Millipore, Kenilworth, NJ, USA); the filtered cells were immersed in 1.6 mL of methanol containing an internal standard (20 µM d-camphor sulfonate) and stored at −80 °C. The DCW in the recovered culture medium was calculated as OD_600_ × conversion factor (g L^−1^ OD_600_^−1^) × volume of the collected medium (L). The conversion factors for each strain were determined by measuring cell weight after collection, using the filter method, and drying at 60 °C for seven days (Table S4). Intracellular metabolites were extracted using methanol/chloroform/water in 640 µL of Milli-Q water and 1.6 mL of chloroform. The mixture was centrifuged (3700× *g*, 4 °C, 20 min), and 250 µL of the supernatant was dispensed into six Eppendorf tubes, dried under vacuum conditions at ambient temperature using a Speed Vac (Thermo Fisher Scientific, Waltham, MA, USA), and stored at −80 °C.

For the isotope dilution method, yeast strain S288C was cultured in SD medium containing 100% [U-^13^C] glucose as the sole carbon source and extracted as described previously [20]. The concentrations of metabolites in the ^13^C labeled sample were measured using the internal standard method after mixing with the unlabeled metabolite (final concentration: 10 µM).

To analyze the industrial strain, unlabeled sample solutions prepared from the industrial strain were mixed with equal amounts of ^13^C-labeled samples from the S288C strains. The mixture was subjected to metabolomic analysis using LC-triple-stage quadrupole MC (LC-MS/MS, LCMS-8060NX, Shimadzu). Metabolome data were obtained using two targeted methods, according to previously reported pentafluorophenylpropyl-LC-MS/MS and ion pair LC-MS/MS methods [20,42]. The multiple reaction monitoring results are shown in Tables S5 and S6. To analyze the industrial strains, metabolite concentrations in the sample solutions were calculated using the following equation:

Metabolite concentration (µM) = (peak area ratio) × (metabolite concentration in the ^13^C-labeled sample derived from S288C).

Herein, the peak area ratio represents the ratio of the peak areas of the unlabeled metabolite signal derived from the industrial strains and the ^13^C-labeled metabolite signal derived from S288C. To analyze the *PDC1*-overexpressing strains, the peak areas of each metabolite signal were normalized by dividing them by the peak area of an internal standard compound.

### 4.4. Calculation of ΔG′ and Multivariate Analysis

The intracellular concentrations of metabolites were calculated based on the following assumptions: The number of cells per OD_600_ × mL was 3.0 × 10^8^ [43]. The yeast cells are spheres with a diameter of 6 µm. The concentrations of phosphate and carbon dioxide were constant at 0.05 mM and 0.046 µM, respectively. The intracellular and extracellular concentrations of glucose and ethanol and the mitochondrial and cytoplasmic metabolite concentrations were identical. The Δ*G*′ of each reaction was calculated using the following formula: Δ*G*′ = Δ*G*′^0^ + *RT lnQ*. The Δ*G*′^0^, *R*, *T*, and *Q* represent the standard Gibbs free energy, gas constant (8.314 J (K mol)^−1^), absolute temperature (303 K), and reaction quotient, respectively. Equilibrium constants (*K*eq) or standard Gibbs free energies (Δ*G*′^0^) for each reaction were obtained from an eQuilibrator and are shown in Table S3 [44].

*t*-SNE and hierarchical clustering were performed using the Scikit-learn package version 1.3.2 in Python 3.8 after Z-score normalization. Hierarchical clustering was performed using the “correlation” and “average” for the distance matrix and cluster calculation, respectively.

### 4.5. Construction of Metabolically Engineered Strains

Plasmid vectors and primers used in this study are listed in Table 1 and Table S7, respectively. Lysogeny broth (10 g L^−1^ Bacto tryptone, 5 g L^−1^ Bacto yeast extract, and 10 g L^−1^ NaCl) was used to culture *Escherichia coli*, and 50 mg L^−1^ ampicillin was added as needed. The growth conditions, DNA techniques, and lithium-acetate method for transformations have been described previously [45,46]. DNA fragments were assembled using the Gibson Assembly Master Mix (New England Biolabs, Ipswich, MA, USA). To construct pGK414-PDC1p-PDC1-PDC1t and pGK424-PDC1p-PDC1-PDC1t, the DNA fragment of the ±1 kb region of the ORF of *PDC1* was amplified from *S. cerevisiae* genomic DNA using the primers PDC1_pGK414_f and PDC1_pGK414_r. Outside regions of the expression cassette of pGK414 and pGK424 [40] were amplified via inverse PCR with primers pGK414_inv_f and pGK414_inv_r (Table S7). The DNA fragment of *PDC1* and the plasmid vector backbone were assembled using the Gibson assembly method. To construct pGK414-TDH3p-PDC1-TDH3t and pGK424-TDH3p-PDC1-TDH3t, three DNA fragments of the *PDC1* ORF, TDH3p, and TDH3t were amplified from *S. cerevisiae* genomic DNA using pATP422-alsLpOp-aldcLIOp as a template. These fragments and the pGK414 or pGK424 backbones were ligated using Gibson assembly. pGK414-noxE and pGK424-noxE were constructed in the same manner using the DNA fragment of a codon-optimized sequence of NADH oxidase (*noxE*) from *L. lactis* [14] synthesized by Thermo Fisher Scientific. Yeast transformation was performed using the Frozen-EZ Yeast Transformation II Kit (Zymo Research, Irvine, CA, USA).

### 4.6. RIM15 Deletion

Genome editing was performed as previously described [41]. pGAL1-RIM15Δ was constructed from the backbone vector (pGAL1-Cas9-tADH1-pGAL1-2BsaI-sgRNAFE(empty)-HDV-tCYC1-CU: purchased from the National Bio-Resource Project (NBRP)) and inserted using the New England Biolabs Golden Gate Assembly Kit (BsaI-HF v2). The insert fragment was designed using the CRISPR Direct webpage (https://crispr.dbcls.jp/) (accessed on 6 August 2022). The prepared plasmid and donor sequences were introduced into YHI030 cells by transformation using a SDGal plate for selection. The donor sequence was prepared using PCR, and the *RIM15* deletion was confirmed using colony PCR.

## Figures and Tables

**Table 1:** Strains and plasmids used in this study.

Strains	Description	Ref.
BY4947	Diploid of S288C (X2180-1A × X2180-1B)	National Bio-Resource Project (NBRP), Japan
Kyokai6	Sake strain	[18]
Kyokai7	Sake strain (NBRC2347)	[18]
Kyokai9	Sake strain (NBRC2377)	[18]
QA23™	Wine strain	Gift from SCETI K.K. Tokyo, Japan
Lalvin EC1118™	Wine strain	Gift from SCETI K.K. Tokyo, Japan
OC-2	Wine strain (NBRC104078)	Purchased from NITE
RedStar	Bread strain (NBRC2375)	Purchased from NITE
NBRC0555	Bread strain	Purchased from NITE
NBRC2043	Bread strain	Purchased from NITE
WLP530	Beer strain	Gift from Suntory Co.
S288C	Identical to BY27002, MATα mal SUC2	NBRP Yeast, Japan
YPH499	MATa ura3-52 lys2-801 ade2-101 trp1-Δ63 his3-Δ200 leu2-Δ1	Stratagene/Agilent Technologies
YSM021 (PDCΔ)	YPH499 pdc1Δ pdc5Δ pdc6Δ MTH1-ΔT(L165F)	[13]
YSM046 (PDCΔ + evolved)	Laboratory-evolved yeast strain derived from the PDCΔ (YSM021) strain	[13]
YHI030	YSM046 [pATP422-alsLpOp-aldcLlOp/pAT425-BDH1]	[13]
YMS001	YPH499 [pGK414]	This study
YMS002	YPH499 [pGK424]	This study
YMS003	YPH499 [pGK414-PDC1]	This study
YMS004	YPH499 [pGK424-PDC1]	This study
YMS005	YPH499 [pGK414-TDH3p_PDC1]	This study
YMS006	YPH499 [pGK424-TDH3p_PDC1]	This study
YMS101	YHI030 rim15Δ	This study
YMS102	YHI030 [pGK423/pGK424]	This study
YMS103	YHI030 [pGK423-ALS/pGK424]	This study
YMS104	YHI030 [pGK423-ALS/pGK424]	This study
YMS105	YHI030 [pGK423-ALS/pGK424-noxE]	This study
YMS106	YHI030_rim15Δ [pGK423-ALS/pGK424-noxE]	This study
Plasmids	Description	Ref.
pGK423	Yeast multi-copy type single-gene expression vector containing PGK1 promoter, PGK1 terminator, 2μ origin, and HIS3 marker	[40]
pGK423-ALS	pGK423, expression of the ALS gene by the PGK1 promoter	This study
pGK414	Yeast low-copy type single-gene expression vector containing PGK1 promoter, PGK1 terminator, CEN/ARS ori, and TRP1 marker	[40]
pGK424	Yeast multi-copy type single-gene expression vector containing PGK1 promoter, PGK1 terminator, CEN/ARS ori, and TRP1 marker	[40]
pGK414-PDC	pGK414, expression of the PDC1 gene by the PDC1 promoter	This study
pGK424-PDC	pGK424, expression of the PDC1 gene by the PDC1 promoter	This study
pGK414-TDH3p-PDC1	pGK414, expression of the PDC1 gene by the TDH3 promoter	This study
pGK424-TDHp3-PDC1	pGK424, expression of the PDC1 gene by the TDH3 promoter	This study
pGK424-noxE	pGK424, expression of the noxE gene by the TDH3 promoter	This study
pGAL1-Cas9-tADH1-pGAL1-2BsaI-sgRNAFE(empty)-HDV-tCYC1-CU	YCp vector, and URA3 marker	[41]
pGAL1-RIM15Δ	pGAL1-Cas9-tADH1-pGAL1-2BsaI-sgRNAFE(RIM15D)-HDV-tCYC1-CU	This study

## Data Availability

The data presented in this study are available upon request from the corresponding author.

## Supplementary Materials

The following supporting information can be downloaded at: https://www.mdpi.com/article/10.3390/ijms242216378/s1.
