# Supplementary material for: Improved 2,3-Butanediol Production Rate of Metabolically Engineered Saccharomyces cerevisiae by Deletion of RIM15 and Activation of Pyruvate Consumption Pathway
Source: Int J Mol Sci. 2023 Nov 15;24(22):16378. doi: 10.3390/ijms242216378 (PMC10671664; doi:10.3390/ijms242216378)
Supplement: Supplementary file 1 [file ijms-24-16378-s001.zip › ijms-2664827 Supplementary Materials.pdf]

Supporting information

Improved 2,3-butanediol production rate of metabolically engineered *Saccharomyces cerevisiae* by deletion of *RIM15* and activation of pyruvate consumption pathway

Masahiko Sugimura, Taisuke Seike, Nobuyuki Okahashi, Yoshihiro Izumi, Takeshi Bamba, Jun Ishi, Fumio Matsuda*

Figure S1 Comparison of metabolites states among 11 industrial yeast strains of yeast by hierarchical clustering

Table S1 Mutations in the open reading frame sequence of *RIM15* detected in the 11 industrial strains

Table S2 Mutations in the amino acid sequence of RIM15p detected in the 11 industrial strains

Table S3 Standard Gibbs free energy change ($\Delta G^{0\prime}$) of metabolic reactions and pathways

Table S4 Dry cell weight of *Saccharomyces cerevisiae* strains

Table S5 Multiple reaction monitoring transitions for ion pair liquid chromatography tandem mass spectrometry

Table S6 Multiple reaction monitoring transitions in liquid chromatography-pentafluorophenylpropyl-tandem mass spectrometry

Table S7 Primers used in this study

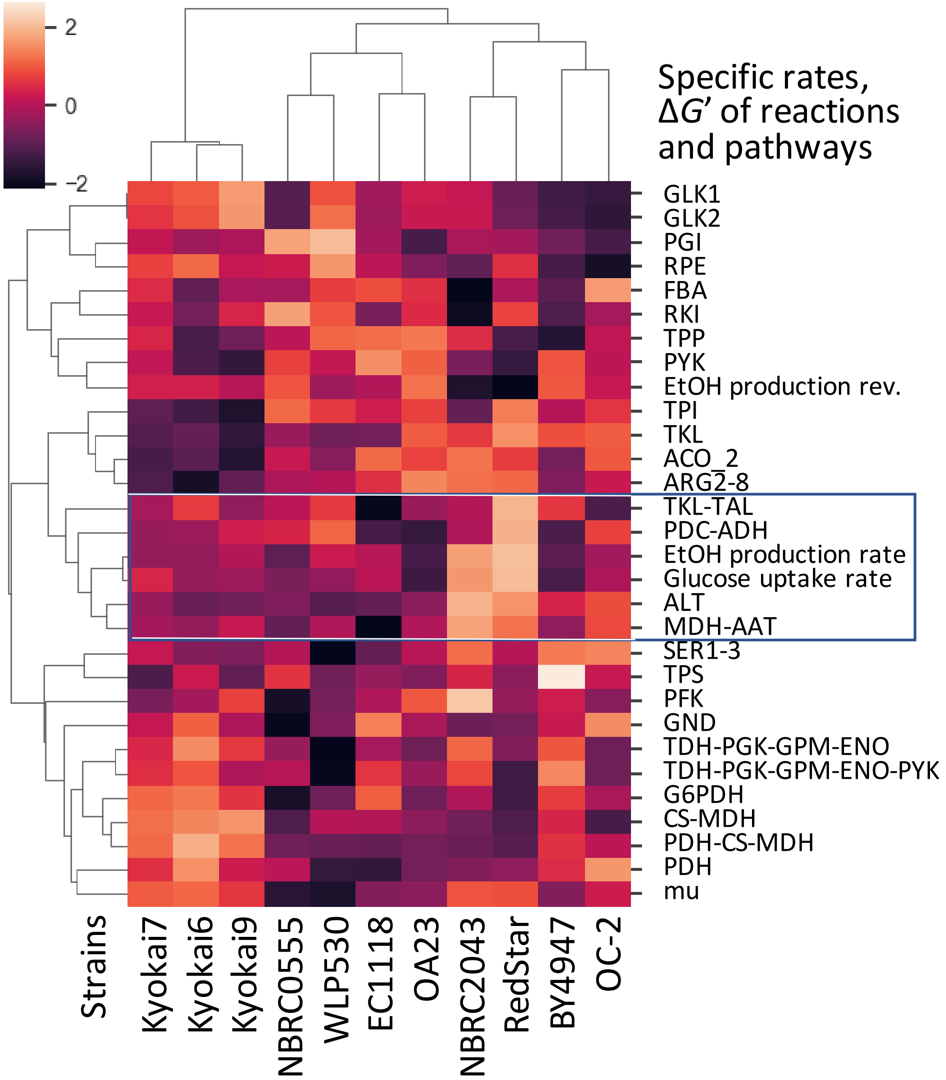

Figure S1. Comparison of metabolites states among 11 industrial yeast strains of yeast by hierarchical clustering. Hierarchical clustering was performed using a data matrix including 26 ΔG′ values and 4 specific rates. The metabolite concentration data was not used because the correlation between the cGMP concentration and ethanol production rate could not be interpreted. The average method and correlation distance matrix were employed.

Table S1 Mutations in the open reading frame sequence of *RIM15* detected from the 11 industrial strains

Strains	Mutation
BY4947	
Kyokai6	C901T, 1042insAATAATAATAAT, C1140T, A1395G, G1869T, G1875A, T1938C, A2016T, G2031A, G2364A, C2517A, C2695T, T2726C, G2832A, C2919T, C2928T, C2941T, A2955G, C2958T, G3160A, T3163G, A3471G, C4191T, G4371A, A4611G, C4722G, G4746A, C4794T, G4803A, A4830T, T4842C, A4869G, C4881T, T5016C, 5055insA, A5214C, C5220T
Kyokai7	C901T, 1042insAATAATAATAAT, C1140T, A1395G, G1869T, G1875A, T1938C, A2016T, G2031A, G2364A, C2517A, C2695T, T2726C, G2832A, C2919T, C2928T, C2941T, A2955G, C2958T, G3160A, T3163G, A3471G, C4191T, G4371A, A4611G, C4722G, G4746A, C4794T, G4803A, A4830T, T4842C, A4869G, C4881T, T5016C, 5055insA, A5214C, C5220T
Kyokai9	C901T, 1042insAATAATAATAAT, C1140T, A1395G, G1869T, G1875A, T1938C, A2016T, G2031A, G2364A, C2517A, C2695T, T2726C, G2832A, C2919T, C2928T, C2941T, A2955G, C2958T, G3160A, T3163G, A3471G, C4191T, G4371A, A4611G, C4722G, G4746A, C4794T, G4803A, A4830T, T4842C, A4869G, C4881T, T5016C, 5055insA, A5214C, C5220T
QA23	T273C, T384C, C393T, A447G, C495T, A795G, T888C, T995A, 1042insAATAATAAT, T1191C, A1395G, G1542A, G1552A, A1821C, A1825T, A2167T, G2469A, G2478A, C2496T, T2910C, C2928T, T3231C, T3345C, T3417C, A3471G, C3537T, C3601T, A3606G, C3789T, C4020T, A4080T, A4119G, C4133T, T4149A, G4371A, C4419T, A4611G, A4650G, A5214C
EC1118	T273C, T384C, C393T, A447G, C495T, A795G, T888C, T995A, 1042insAATAATAAT, T1191C, A1395G, G1542A, G1552A, A1821C, A1825T, A2167T, G2469A, G2478A, C2496T, T2910C, C2928T, T3231C, T3345C, T3417C, A3471G, C3537T, C3601T, A3606G, C3789T, C4020T, A4080T, A4119G, C4133T, T4149A, C4189G, G4371A, C4419T, A4611G, A4650G, A5214C
OC-2	T273C, T384C, G392A, C393T, A447G, C495T, C667T, A795G, T888C, T995A, 1042insAATAATAAT, T1191C, A1395G, G1542A, G1552A, A1821C, A1825T, A2167T, G2469A, G2478A, C2496T, T2910C, C2928T, T3231C, T3345C, T3417C, A3471G, C3537T, C3601T, A3606G, C3789T, C4020T, A4080T, A4119G, C4133T, T4149A, G4371A, C4419T, A4611G, A4650G
RedStar	T273C, T384C, C393T, A447G, C495T, G538A, A795G, T888C, 1042insAATAATAAT, T1191C, A1395G, A1821C, A1825T, A2167T, G2469A, G2478A, C2496T, T2910C, C2928T, T3231C, A3296G, T3345C, T3417C, A3471G, C3537T, C3601T, A3606G, C3789T, C4020T, A4080T, A4119G, C4133T, T4149A, G4371A, C4419T, A4611G, A4650G, A5214C

NBRC0555	A35G, T273C, T384C, G392A, C393T, A447G, C495T, G538A, T888C, 1042insAATAATAAT, T1191C, A1395G, G1810C, A1821C, A1825T, C2046A, A2167T, G2469A, G2478A, C2496T, C2643A, T2910C, C2928T, T3231C, T3345C, T3417C, A3471G, C3537T, C3601T, A3606G, C3789T, C4020T, A4080T, A4119G, C4133T, T4149A, G4371A, C4419T, A4611G, A4650G, A5214C
NBRC2043	T273C, T384C, C393T, A447G, C495T, G538A, A795G, T888C, 1042insAATAATAAT, T1191C, A1395G, A1821C, A1825T, A2167T, G2469A, G2478A, C2496T, T2910C, C2928T, T3231C, A3296G, T3345C, T3417C, A3471G, C3537T, C3601T, A3606G, C3789T, C4020T, A4080T, A4119G, C4133T, T4149A, C4191T, G4371A, C4419T, A4611G, A4650G, A5214C
WLP530	T384C, C393T, A447G, C495T, A795G, T888C, 1042insAATAATAATAATAATAAT, A1063T, T1191C, A1395G, C1622T, A1821C, A1825T, A2167T, G2469A, G2478A, C2495T, C2496T, T2910C, C2928T, T3231C, A3471G, A4611G, C4722G, G4746A, C4794T, G4803A, A4830T, A4869G, C4881T, C5107G, A5214C, C5220T

Table S2 Mutations in the amino acid sequence of RIM15p detected from the 11 industrial strains

	BY4947	Kyokai6	Kyokai7	Kyokai9	QA23	EC1118	OC-2	RedStar	NBRC0555	NBRC2043	WLP530
Q12R									+		
S131N							+		+		
D180N								+	+	+	
R301C		+	+	+							
L332H					+	+	+				
N355T											+
E518K					+	+	+				
P541L											+
G604R									+		
E607D					+	+	+	+	+	+	+
T609S					+	+	+	+	+	+	+
T723S					+	+	+	+	+	+	+
A832V											+
H981Y		+	+	+							
A1054T		+	+	+							
S1055A		+	+	+							
K1099R								+		+	
T1378I					+	+	+	+	+	+	
P1397A						+					
A1686fs		+	+	+							
Q1703E											+
K1738N					+	+		+	+	+	+

Table S3 Standard Gibbs free energy change ($\Delta G'^{0}$) of metabolic reactions and pathways

Reaction or pathway	Stoichiometry	$\Delta G'^{0}$ (kJ/mol)
GLK	Glc+ATP<->G6P+ADP	-20.4
PGI	G6P<->F6P	2.6
PFK	F6P+ATP<->FBP+ADP	-16.7
FBA	FBP<->DHAP+GAP	20.1
TPI	DHAP<->GAP	5.6
ENO	2PG<->PEP	3.8
PYK	PEP+ADP<->Pyr+ATP	-25
PDH	Pyr+NAD^++CoASH<->Ac-CoA+NADH+CO_2	-42.3
G6PDH	G6P+$NADP^+$<->6PG+NADPH	-2.4
GND	6PG+$NADP^+$<->Ru5P+NADPH+CO_2	2.3
RKI	Ru5P<->R5P	-2.1
RPE	Ru5P<->Xu5P	-3.4
TKL	R5P+Xu5P<->S7P+GAP	-3.8
ACO1	Citric acid<->Isocitric acid	6.8
ACO2	Citric acid<-> cis-Aconitic acid	8.5
ACO3	cis-Aconitic acid <->Isocitric acid	-1.6
IDH	Isocitric acid+NAD^++<->2KG+NADH+CO_2	-2.6
ALT	Pyr+Glu<->Ala+2KG	-0.2
TDH-PGK-GPM-ENO	GAP+NAD^++ADP+Pi<->PEP+NADH+ATP	-17.7
TDH-PGK-GPM-ENO-PYR	GAP+NAD^++2 ADP+Pi<->Pyr+NADH+2 ATP	-42.7
CS-MDH	Malate+NAD^++Ac-CoA+H_2O<->NADH+CoA-SH+Citric acid	-12.3
TKL-TAL	R5P+2 Xu5P<->2 F6P+GAP	-15.1
SER	3PG+NAD^++Glu+H2O<->Ser+NADH+2KG+Pi	9.6
MDH-AAT	Malic acid+NAD^++Glu<->Asp+NADH+2KG	-10.6
ARG2,5,6,7,8	2 Glu+Ac-CoA+ATP+NADPH<->Ac-Orn+CoA-SH+ADP+$NADP^+$+Pi+2KG	-2.3
ARG1,3,4	Orn+Carbamoyl-P+ATP+Asp<->Arg+3 Pi+AMP+Fumalic acid	-39.5
ADE1,2,4,5,6,7,8,13,16,17	2 Gln+CO_2+Asp+5 ATP+R5P+2 Ser+2 $NADP^+$+2 H_2O<->IMP+2 Glu+6 Pi+4 ADP+AMP+Fumaric acid+Gly+2 NADPH	-153.2
URA-1,2,3,4,5,6,10	Glu+R5P+4 ATP+Asp+NAD^++3 H_2O<->UDP+4 Pi+3 ADP+AMP+Glu+NADH	-108.6
TKL-ARO1,2,3,4,8-PHA	F6P+G3P+2 PEP+ATP+Glu+NADPH<->Phe+Xu5P+ADP+4 pi+CO2+H_2O+2KG+$NADP^+$	-486.5
PDC-ADH	Pyr+NADH+<->EtOH+NAD^++CO_2	-39.7

Table S4 Dry cell weight of *S. cerevisiae* strains

Strains	Dry cell weight (g DCW $(OD_{600}\ L)^{-1}$)
BY4947	0.295
Kyokai 6	0.317
Kyokai 7	0.281
Kyokai 9	0.344
QA23	0.316
EC1118	0.328
OC-2	0.314
RedStar	0.326
NBRC2043	0.315
NBRC0555	0.353
WLP530	0.309

Table S5 MRM transitions for ion pair LC-MS/MS

Compounds	Precursor ion (*m/z*)	Product ion (*m/z*)	Target Q1 Pre Bias (V)	Target Collision Energy (V)	Target Q3 Pre Bias (V)	Ionazation polality
10-Camphor sulfonic acid (IS)	231.3	79.9	36	31	19	negative
Argininosuccinic acid	290.9	70.2	-14	-37	-14	positive
[U-^{13}C]Argininosuccinic acid	300.9	74.2	-14	-37	-14	positive
Fumaric acid	115.2	71.1	11	10	16	negative
[U-^{13}C]Fumaric acid	119.2	74.1	11	10	16	negative
Arginine	174.9	70.2	-12	-24	-28	positive
[U-^{13}C]Arginine	180.9	74.2	-12	-24	-28	positive
Ornithine	133.1	70.1	-34	-22	-28	positive
[U-^{13}C]Ornithine	138.1	74.1	-34	-22	-28	positive
Citrulline	175.9	70.2	-12	-24	-15	positive
[U-^{13}C]Citrulline	181.9	74.2	-12	-24	-15	positive
N-Acetylornithine	174.9	70.2	-15	-26	-27	positive
[U-^{13}C]*N*-Acetylornithine	181.9	74.2	-15	-26	-27	positive
N-Acetylglutamic acid	189.9	130.1	-15	-13	-12	positive
[U-^{13}C]*N*-Acetylglutamic acid	196.9	135.1	-15	-13	-12	positive
Glutamic acid	147.8	84.1	-10	-17	-20	positive
[U-^{13}C]Glutamic acid	152.8	88.1	-10	-17	-20	positive
2-Ketoglutaric acid	145.1	101.0	13	10	17	negative
[U-^{13}C]2-Ketoglutaric acid	150.1	105.0	13	10	17	negative
Succinic acid	117.4	73.0	11	11	15	negative
[U-^{13}C]Succinic acid	121.4	76.0	11	11	15	negative
Malic acid	133.2	115.0	13	15	22	negative
[U-^{13}C]Malic acid	137.2	119.0	13	15	22	negative
Citric acid	191.2	111.0	12	11	19	negative
[U-^{13}C]Citric acid	197.2	116.0	12	11	19	negative
cis-Aconitic acid	173.2	85.0	19	12	20	negative
[U-^{13}C]*cis*-Aconitic acid	179.2	89.0	19	12	20	negative
Isocitric acid	191.2	111.1	19	13	10	negative
[U-^{13}C]Isocitric acid	197.2	116.1	19	13	10	negative
Aspartic acid	134.0	74.0	-12	-16	-26	positive
[U-^{13}C]Aspartic acid	138.0	77.0	-12	-16	-26	positive

Guanine	152.0	135.0	-13	-22	-20	positive
[U-^{13}C]Guanine	157.0	140.0	-13	-22	-20	positive
Guanosine	283.9	152.0	-20	-15	-20	positive
[U-^{13}C]Guanosine	293.9	157.0	-20	-15	-20	positive
GMP	363.8	152.1	-10	-17	-14	positive
[U-^{13}C]GMP	373.8	157.1	-10	-17	-14	positive
Deoxyguanosine	267.9	152.1	-10	-11	-15	positive
[U-^{13}C]Deoxyguanosine	277.9	157.1	-10	-11	-15	positive
dGMP	348.0	152.2	-10	-14	-15	positive
[U-^{13}C]dGMP	358.0	157.2	-10	-14	-15	positive
Adenine	136.2	119.0	-10	-26	-19	positive
[U-^{13}C]Adenine	141.2	124.0	-10	-26	-19	positive
Adenosine	268.0	136.0	-13	-19	-23	positive
[U-^{13}C]Adenosine	278.0	141.0	-13	-19	-23	positive
AMP	348.0	136.1	-10	-21	-13	positive
[U-^{13}C]AMP	358.0	141.1	-10	-21	-13	positive
Deoxyadenosine	251.9	136.0	-20	-15	-20	positive
[U-^{13}C]Deoxyadenosine	261.9	141.0	-20	-15	-20	positive
dAMP	332.1	136.1	-16	-17	-14	positive
[U-^{13}C]dAMP	342.1	141.1	-16	-17	-14	positive
Thymine	127.2	110.1	-18	-21	-17	positive
[U-^{13}C]Thymine	132.2	115.1	-18	-21	-17	positive
Thymidine	243.1	127.2	-12	-11	-13	positive
[U-^{13}C]Thymidine	253.1	132.2	-12	-11	-13	positive
TMP	320.9	195.1	20	16	21	negative
[U-^{13}C]TMP	330.9	200.0	20	16	21	negative
dTMP	322.8	81.0	-12	-17	-18	positive
[U-^{13}C]dTMP	332.8	85.0	-12	-17	-18	positive
Cytosine	111.9	95.1	-10	-21	-20	positive
[U-^{13}C]Cytosine	116.9	99.1	-10	-21	-20	positive
Cytidine	243.8	112.1	-12	-12	-11	positive
[U-^{13}C]Cytidine	252.8	116.1	-12	-12	-11	positive
CMP	324.0	112.1	-12	-14	-11	positive
[U-^{13}C]CMP	333.0	116.1	-12	-14	-11	positive
Deoxycytidine	227.9	112.1	-11	-10	-11	positive

[U-^{13}C]Deoxycytidine	236.9	116.1	-11	-10	-11	positive
dCMP	307.8	112.1	-14	-12	-11	positive
[U-^{13}C]dCMP	316.8	116.1	-14	-12	-11	positive
Uracil	111.3	42.1	14	15	15	negative
[U-^{13}C]Uracil	115.3	44.0	14	15	15	negative
Uridine	245.0	113.1	-12	-11	-11	positive
[U-^{13}C]Uridine	254.0	117.1	-12	-11	-11	positive
UMP	325.1	97.2	-12	-17	-21	positive
[U-^{13}C]UMP	334.1	97.2	-12	-17	-21	positive
Deoxyuridine	229.0	113.1	-11	-11	-18	positive
[U-^{13}C]Deoxyuridine	238.0	117.1	-11	-11	-18	positive
Hypoxanthine	137.0	110.1	-12	-24	-22	positive
[U-^{13}C]Hypoxanthine	142.0	115.0	-12	-24	-22	positive
Inosine	268.9	137.1	-13	-11	-13	positive
[U-^{13}C]Inosine	278.9	142.1	-13	-11	-13	positive
IMP	349.1	137.1	-10	-15	-13	positive
[U-^{13}C]IMP	359.1	142.1	-10	-15	-13	positive
Xanthine	151.3	108.0	14	17	21	negative
[U-^{13}C]Xanthine	156.3	112.0	14	17	21	negative
Xanthosine	285.0	153.1	-14	-11	-15	positive
[U-^{13}C]Xanthosine	295.0	158.1	-14	-11	-15	positive
cAMP	329.8	136.1	-15	-26	-13	positive
[U-^{13}C]cAMP	339.8	141.1	-15	-26	-13	positive
cGMP	345.8	152.1	-16	-20	-15	positive
[U-^{13}C]cGMP	355.8	157.1	-16	-20	-15	positive
Orotic acid	155.2	111.0	14	11	22	negative
[U-^{13}C]Orotic acid	160.2	115.0	14	11	22	negative
GSH	307.8	76.1	-14	-27	-28	positive
[U-^{13}C]GSH	317.8	78.1	-14	-27	-28	positive
GSSG	612.9	231.1	-22	-38	-22	positive
[U-^{13}C]GSSG	632.9	239.1	-22	-38	-22	positive
GABA	104.0	87.1	-10	-14	-20	positive
[U-^{13}C]GABA	108.0	91.1	-10	-14	-20	positive

Table S6 MRM transitions for the LC-PFPP-MS/MS

Compounds	Precursor ion (*m/z*)	Product ion (*m/z*)	Target Q1 Pre Bias (V)	Target Collision Energy (V)	Target Q3 Pre Bias (V)	Ionazation polality
10-Camphor sulfonic acid (IS)	231.3	79.9	15	31	15	negative
[U-^{13}C]Lysine	151.2	151.2	16	12	29	negative
Arginine	173.1	131.2	21	14	25	negative
[U-^{13}C]Arginine	179.1	136.2	21	14	25	negative
Histidine	154.1	93.1	14	17	18	negative
[U-^{13}C]Histidine	160.1	98.1	14	17	18	negative
Glycine	74.1	74.1	12	10	14	negative
[U-^{13}C]Glycine	76.1	76.1	12	10	14	negative
Serine	104.1	74.1	29	14	29	negative
[U-^{13}C]Serine	107.1	76.1	29	14	29	negative
Asparagine	131.0	113.2	13	12	21	negative
[U-^{13}C]Asparagine	135.0	117.2	13	12	21	negative
Alanine	88.1	88.1	24	15	16	negative
[U-^{13}C]Alanine	91.1	91.1	24	15	16	negative
Glutamine	145.0	127.1	17	14	24	negative
[U-^{13}C]Glutamine	150.0	132.1	17	14	24	negative
Threonine	118.0	74.1	11	13	30	negative
[U-^{13}C]Threonine	122.0	76.1	11	13	30	negative
Cysteine	120.0	33.1	12	12	12	negative
[U-^{13}C]Cysteine	123.0	33.1	12	12	12	negative
Methionine	148.1	47.1	13	12	19	negative
[U-^{13}C]Methionine	153.1	48.1	13	12	19	negative
Tyrosine	180.0	119.1	22	14	30	negative
[U-^{13}C]Tyrosine	189.0	127.1	22	14	30	negative
Glutamic acid	146.0	102.1	17	15	18	negative
[U-^{13}C]Glutamic acid	151.0	106.1	17	15	18	negative
Aspartic acid	132.1	88.2	13	12	17	negative
[U-^{13}C]Aspartic acid	136.1	91.2	13	12	17	negative
Phenylalanine	164.0	147.1	10	14	28	negative

[U-^{13}C]Phenylalanine	173.0	156.1	10	14	28	negative
G6P	259.0	97.1	30	16	18	negative
[U-^{13}C]G6P	265.0	97.1	30	16	18	negative
R5P	229.0	97.1	29	13	18	negative
[U-^{13}C]R5P	234.0	97.1	29	13	18	negative
S7P	289.0	97.1	18	21	18	negative
[U-^{13}C]S7P	296.0	97.1	18	21	18	negative
F6P	259.0	97.1	12	15	18	negative
[U-^{13}C]F6P	265.0	97.1	12	15	18	negative
G1P	258.9	79.1	11	24	14	negative
[U-^{13}C]G1P	264.9	79.1	11	24	14	negative
GAP	169.0	97.1	20	9	18	negative
[U-^{13}C]GAP	172.0	97.1	20	9	18	negative
E4P	198.9	97.1	24	9	19	negative
[U-^{13}C]E4P	202.9	97.1	24	9	19	negative
Ru5P	229.0	97.1	28	11	19	negative
[U-^{13}C]Ru5P	234.0	97.1	28	11	19	negative
Xu5P	229.0	97.1	28	11	19	negative
[U-^{13}C]Xu5P	234.0	97.1	28	11	19	negative
Pyruvic acid	87.0	43.0	24	12	16	negative
[U-^{13}C]Pyruvic acid	90.0	45.0	24	12	16	negative
CMP	322.0	79.1	14	40	30	negative
[U-^{13}C]CMP	331.0	110.0	14	40	21	negative
NAD	662.0	540.0	30	13	40	negative
[U-^{13}C]NAD	683.0	555.0	30	13	40	negative
DHAP	169.0	97.1	21	9	18	negative
[U-^{13}C]DHAP	172.0	97.1	21	9	18	negative
UMP	322.9	79.1	20	40	30	negative
[U-^{13}C]UMP	331.9	111.0	20	40	21	negative
GMP	361.9	79.1	23	35	30	negative
[U-^{13}C]GMP	371.9	79.1	23	35	30	negative
TMP	320.9	195.1	20	16	21	negative
[U-^{13}C]TMP	330.9	204.1	20	16	21	negative
AMP	346.0	79.1	15	26	30	negative
[U-^{13}C]AMP	356.0	79.1	15	26	30	negative

cGMP	343.9	150.1	21	24	29	negative
[U-^{13}C]cGMP	353.9	155.0	21	24	29	negative
cAMP	327.9	134.2	14	24	27	negative
[U-^{13}C]cAMP	337.9	139.2	14	24	27	negative
CDP	402.0	158.9	18	27	29	negative
[U-^{13}C]CDP	411.0	158.9	18	27	29	negative
6PG	275.0	97.1	12	17	18	negative
[U-^{13}C]6PG	281.0	97.1	12	17	18	negative
GDP	441.9	159.1	28	27	30	negative
[U-^{13}C]GDP	451.9	159.1	28	27	30	negative
UDP	403.0	159.1	18	26	29	negative
[U-^{13}C]UDP	412.0	159.1	18	26	29	negative
3PG+2PG	185.1	97.1	21	13	18	negative
[U-^{13}C]3PG+2PG	188.1	97.1	21	13	18	negative
NADP	742.2	619.9	34	15	32	negative
[U-^{13}C]NADP	763.2	634.9	34	15	32	negative
FBP	339.1	97.1	15	19	18	negative
[U-^{13}C]FBP	345.1	97.1	15	19	18	negative
NADH	664.0	408.0	28	34	27	negative
[U-^{13}C]NADH	685.0	418.0	28	34	27	negative
ADP	425.9	159.0	19	26	30	negative
[U-^{13}C]ADP	435.9	159.0	19	26	30	negative
TDP	401.0	159.0	18	24	30	negative
[U-^{13}C]TDP	411.0	159.0	18	24	30	negative
PEP	167.1	79.1	10	11	30	negative
[U-^{13}C]PEP	170.1	79.1	10	11	30	negative
CTP	481.9	159.0	22	30	29	negative
[U-^{13}C]CTP	490.9	159.0	22	30	29	negative
GTP	521.9	159.0	24	35	30	negative
[U-^{13}C]GTP	531.9	159.0	24	35	30	negative
UTP	482.9	159.0	30	32	30	negative
[U-^{13}C]UTP	491.9	159.0	30	32	30	negative
ATP	505.9	159.1	22	30	29	negative
[U-^{13}C]ATP	515.9	159.1	22	30	29	negative
TTP	481.1	159.0	21	30	29	negative

[U-^{13}C]TTP	491.1	159.0	21	30	29	negative
NADPH	744.2	408.2	34	38	29	negative
[U-^{13}C]NADPH	765.2	418.2	34	38	29	negative
Succinyl coenzyme A	866.0	408.0	40	41	28	negative
[U-^{13}C] Succinyl coenzyme A	891.0	418.0	40	41	28	negative
Acetyl coenzyme A	808.0	408.0	36	37	19	negative
[U-^{13}C] Acetyl coenzyme A	831.0	418.0	36	37	19	negative
Carbamoyl phosphate	140	97	21	9	18	negative
[U-^{13}C] Carbamoyl phosphate	141	97	21	9	18	negative
PRPP	388.85	290.95	18	14	18	negative
[U-^{13}C] PRPP	393.85	295.95	18	14	18	negative
CoA-SH	766.1	408.1	20	30	27	negative
[U-^{13}C] CoA-SH	787	418	20	30	27	negative
Trehalose	341.1	179.2	16	14	11	negative
[U-^{13}C] Trehalose	353.1	185.2	16	14	11	negative
UDP-Glc	565.05	323.05	22	27	15	negative
[U-^{13}C] UDP-Glc	580.05	329.05	22	27	15	negative
T6P	421.05	79.1	19	26	30	negative
[U-^{13}C] T6P	433.05	79.1	19	26	30	Negative

Table S7 Primers used in this study

Name	Sequence	Purpose
pGK423_als_r	TTGTCTGGCATGTGTCGACGCTAGCGTTTTATATTTG	pGK423-ALS, Inverse PCR of pGK423
pGK423_als_f	AATTCTACTAACCCGGGTCTAGAGAATTCAGATCTG	pGK423-ALS, Inverse PCR of pGK423
ALS_pGK423_f	TAGTGGATCCATGCCAGACAAAAAGTATTATGGTGCC	pGK423-ALS, Cloning of ALS ORF
ALS_pGK423_r	TAGACCCGGGTTAGTAGAATTGGTCTGGCAACATAGTTTTACC	pGK423-ALS, Cloning of ALS ORF
pGK414_inv_r	ACCGTCATCACCGAAACGC	pGK414-PDC and pGK424-PDC, Inverse PCR of pGK414, 424
pGK414_inv_f	TCACAGCTTGTCTGTAAGCGGATG	pGK414-PDC and pGK424-PDC, Inverse PCR of pGK414, 424
PDC1_pGK414_f	CGCGTTTCGGTGATGACGGTCTAGCTGTCCTCGTTGAACATAGG	pGK414-PDC and pGK424-PDC, Cloning of PDC1 ORF
PDC1_pGK414_r	CCGCTTACAGACAAGCTGTGAGAAACCACACTGTTTAAACAGTGTTCCTTAATC	pGK414-PDC and pGK424-PDC, Cloning of PDC1 ORF
PDC1_TDH3p	AAACACACATAAACAAACAAAATGTCTGAAATTACTTTGGGTAAATATTTGTTCGAAAG	pGK414-TDH3p-PDC and pGK424-TDH3p-PDC, Cloning of TDH3p ORF
PDC1_TDH3t	CAAGATTTAAAGTAAATTCACTTATTGCTTAGCGTTGGTAGCAGC	pGK414-TDH3p-PDC and pGK424-TDH3p-PDC, Cloning of TDH3p ORF
TDH3prom_f_pGK	CGCGTTTCGGTGATGACGGTTCATTATCAATACTGCCATTTCAAAGAATACG	pGK414-TDH3p-PDC and pGK424-TDH3p-PDC
TDH3prom_r	TTTGTTTGTTTATGTGTGTTTATTCGAAACTAAGTTC	pGK414-TDH3p-PDC and pGK424-TDH3p-PDC
TDH3term_f	GTGAATTTACTTTAAATCTTGCATTTAAATAAATTTTCTTTTTATAGC	pGK414-TDH3p-PDC and pGK424-TDH3p-PDC

TDH3term_r_pGK	CGCTTACAGACAAGCTGTGATTAATTAAATCCTGGCGGAAAAAATTCATTTG	pGK414-TDH3p-PDC and pGK424-TDH3p-PDC
noxE_prom	ATGAAGATCGTTGTCATCGGTACTAATCATG	pGK424-noxE
noxE_term	CTACTTGGCATTCAAAGCAGCAACAG	pGK424-noxE
noxE_prom_TDH3	AACACACATAAACAAACAAAATGAAGATCGTTGTCATCGGTACTAATCATG	pGK424-noxE
noxE_term_TDH3	AAGATTTAAAGTAAATTCACctaCTTGGCATTCAAAGCAGCAACAG	pGK424-noxE
oTS1407	GGAGGAATGTCTGATGAGTCCGTGAGGACGAAACGAGTAAGCTCGTCACATTCAAAACGTCACGTCG	pGAL1-RIM15Δ insert fragment forward
oTS1408	AAACCGACGTGACGTTTTGAATGTGACGAGCTTACTCGTTTCGTCCTCACGGACTCATCAGACATTC	pGAL1-RIM15Δ insert fragment reverse
oTS1409	TTTCTCTTGCCTCATTTGATAGAATAGATAAGCCCAGTAGAGGAAGACAGTAAATTTTAA	pGAL1-RIM15Δ donor sequence forward
oTS1410	CAAAGTTTTTATTCAGTTATTTTTTTAATTATCTTTATCTTAAAATTTACTGTCTTCCT	pGAL+A32:C76IM15Δ donor sequence reverse
